# A novel salt-tolerant strain *Trichoderma atroviride* HN082102.1 isolated from marine habitat alleviates salt stress and diminishes cucumber root rot caused by *Fusarium oxysporum*

**DOI:** 10.1186/s12866-022-02479-0

**Published:** 2022-03-01

**Authors:** Chongyuan Zhang, Weiwei Wang, Yihui Hu, Zhongpin Peng, Sen Ren, Ming Xue, Zhen Liu, Jumei Hou, Mengyu Xing, Tong Liu

**Affiliations:** 1 Key Laboratory of Green Prevention and Control of Tropical Diseases and Pests, Hainan University, School of Plant Protection, Ministry of Education, Haikou, Hainan 570228 PR China; 2 Key Laboratory of Genetics and Germplasm Innovation of Tropical Special Forest Trees and Ornamental Plants, Hainan University, Ministry of Education, Haikou, Hainan 570228 PR China; 3 Key Laboratory of Germplasm Resources of Tropical Special Ornamental Plants of Hainan Province, College of Forestry, Haikou, Hainan 570228 PR China; 4 Engineering Centre of Agricultural Microbial Preparation Research and Development of Hainan, Hainan University, Haikou, Hainan 570228 PR China

**Keywords:** Biological control, Cucumber root rot, Growth promotion, Salt stress, *Trichoderma atroviride*

## Abstract

**Background:**

Salt stress threaten the growth of plants, and even aggravate plant disease. In this article, salt-tolerant *Trichoderma* strain was isolated, and its potential to alleviate salt stress and diminish cucumber root rot caused by *Fusarium oxysporum* was evaluated.

**Results:**

Twenty-seven *Trichoderma* isolates were isolated from samples of sea muds and algae collected from the South Sea of China. Among these, the isolate HN082102.1 showed the most excellent salt tolerance and antagonistic activity against *F. oxysporum* causing root rot in cucumber and was identified as *T. atroviride*. Its antagonism ability may be due to mycoparasitism and inhibition effect of volatile substances. The application of *Trichoderma* mitigated the adverse effects of salt stress and promoted the growth of cucumber under 100 mM and 200 mM NaCl, especially for the root. When *T. atroviride* HN082102.1 was applied, root fresh weights increased by 92.55 and 84.86%, respectively, and root dry weights increased by 75.71 and 53.31%, respectively. Meanwhile, the application of HN082102.1 reduced the disease index of cucumber root rot by 63.64 and 71.01% under 100- and 0-mM saline conditions, respectively, indicating that this isolate could inhibit cucumber root rot under salt stress.

**Conclusions:**

This is the first report of salt-tolerant *T. atroviride* isolated from marine habitat showing antagonistic activity to *F. oxysporum*, and the results provide evidence for the novel strain *T. atroviride* HN082102.1 in alleviating salt stress and diminishing cucumber root rot, indicating that *T. atroviride* strain HN082102.1 can be used as biological control agent in saline alkali land.

## Background

Salt stress affects almost every aspect of the physiology and biochemistry of plants, thereby limiting plant growth and productivity [1], and even the morphological development of individual plant [2]. Under salt stress, the accumulation of Na^+^ and Cl^−^ in cells significantly decreases the content of glycolipids and unsaturated fatty acids, increases the content of saturated fatty acids in the thylakoid membrane, and inhibits the activity of photosynthesis-related enzymes such as Rubisco and PEP carboxylase, thereby disrupting the photosynthetic capacity [3]. Also, reactive oxygen species (ROS) showing strong oxidation ability accumulates, and damages unsaturated fats, proteins, nucleic acids, and other biomolecules [4]. What’s more, salt stress exacerbates the degree of membrane lipid peroxidation, leading to the destruction of membrane integrity, massive leakage of electrolytes and some small organic molecules, and disruption of the material exchange balance within cell [5]. Therefore, salt stress makes plants more vulnerable to a variety of threats including pathogens. Many studies have reported that salt stress can aggravate the effects of pathogenic diseases on plants, such as *Phytophthora capsica* [6], *Fusarium oxysporum* [7], *Alternaria solani* [8], and so on. In China, there is about 2.6 × 10^7^ ha of saline-alkali land [9]. The salt content of saline-alkali land is greater than 0.6%, and it is difficult for plants to grow on this type of land. Therefore, it is important to improve the utilization of saline-alkali soil in order to increase cultivated land area and ensure ecological security.

Root rot caused by *F. oxysporum* is an important soil-borne disease in cucumber. Various control methods are used to avoid losses in cucumber yield due to root rot. Physical methods such as sunlight or ultraviolet radiation [10] and chemical methods [11] are commonly used. Although the chemical fungicides are very effective in controlling plant fungal diseases, they also bring many problems, such as the fungicide-resistance of pathogens and the ecological security [12]. So, researchers are now trying to use environmentally safe methods to control root rot, such as biological agents [13].

*Trichoderma* species are widely distributed in nature worldwide, and have been successfully used as biofertilizers, biopesticides and bioremediation agents [14, 15]. It is reported that *Trichoderma* played an important role in reducing the adverse effects of salt stress in the desert shrub *Ochradenus baccatus* [16]. *Trichoderma* can be used to control plant disease mainly due to their antagonistic and mycoparasitic activity [17–19]. Also, they can enhance nutrients absorption of plant [20] and promote the production of numerous biochemical elicitors, including all kinds of peroxidases (PODs), chitinase, β-1,3-glucanase, lipoxygenase-pathway hydroperoxide lyase, and compounds such as phytoalexins and phenols, thereby promoting growth and enhancing biotic and abiotic stress tolerance of plants [21–23]. So, the aim of the present study was to determine whether *Trichoderma* could alleviate the negative effects of salt stress and diminish root rot in cucumber.

## Methods

### Identification of the pathogen of cucumber root rot

Diseased cucumber plants with typical symptoms of root rot or damping-off (rotted roots, wilted leaves, leaves cannot be spread normally, leaf tip chlorosis) were collected from cucumber fields in Hainan province, immediately placed into plastic bags and stored in iceboxes. The pathogenic fungus was isolated by the tissue separation method, and the pathogenicity of the purified fungus was determined according to Koch’s postulates [24]. The isolated pathogen was identified through morphological characterization according to the previous descriptions of *F. oxysporum* [25] and molecular analysis depending on the internal transcribed spacer (*ITS*) regions of rDNA.

### Isolation of Trichoderma

Samples of forty-six sea muds and three algae were collected from marine habitats in three districts of Hainan Province, China: Dongfang City (18°43′–9°18′N, 108°36′–109°07′E), Sanya City (18°09′–18°37′N, 108°56′–109°48′E), and Lingao County (19°34′–20°02′N, 109°3′–109°53′E). Samples were placed in sterile polyethylene bags, stored in an icebox and transported to the laboratory as soon as possible. *Trichoderma* spp. were isolated from the sea mud samples using a dilution method with a modified potato dextrose agar medium (mPDA) (200 g/L potato, 20 g/L glucose, 18 g/L agar, 0.1 g/L streptomycin, 0.3 g/L chloramphenicol, 0.02 g/L rose bengal) [26]. The algae samples were washed thoroughly in running tap water, then in deionized water, and cut into 10 mm × 10 mm fragments with a flame-sterilized razor blade. The fragments were surface sterilized by 75% ethanol for 30 s, 8% NaClO solution for 1 min, and 75% ethanol for 30 s, rinsed with sterile double-distilled water for five times to remove excess chemical sterilizing agents, placed on mPDA in Petri dishes, and incubated at 28 °C for 48 h. The putative *Trichoderma* isolates were selected, and purified by single spore isolation [27], then preserved at 4 °C at the Engineering Center of Agricultural Microbial Preparation Research and Development of Hainan (Hainan University) for subsequent research.

### Screening of Trichoderma with salt tolerance and antagonistic activity

To assess salt tolerance, all *Trichoderma* isolates isolated above were cultured on PDA amended with various concentrations of NaCl (3, 5, 7, and 9% [w/v]), and incubated at 28 °C. The diameters of colonies on NaCl-amended media were recorded on the 3rd, 5th, and 7th day.

To evaluate the antagonistic activity against the pathogen of cucumber root rot, all *Trichoderma* isolates isolated above which could grow on PDA medium containing 9% NaCl and *F. oxysporum* HGL.1 were dual cultured on PDA in the presence or absence of 100 mM NaCl salt stress [28]. The plates were incubated at 28 °C for 7 days, and the radial growth radius of HGL.1 were measured.

### Identification of salt-tolerant Trichoderma

The morphological and molecular identification of the *Trichoderma* strain showing salt tolerance and antagonistic activity were carried out. For morphological identification, hyphal morphology and colony growth patterns of salt-tolerant *Trichoderma* isolate were observed at 72, 96, 120, 144, and 168 h post inoculation on PDA, synthetic low nutrient agar (SNA: 1.0 g/L KH_2_PO_4_, 10.5 g/L KCl, 1.0 g/L KNO_3_, 0.5 g/L MgSO_4_, 0.2 g/L dextrose, 0.2 g/L sucrose, 18 g/L agar), and corn meal dextrose agar (CMD: 4% [w/v] cornmeal + 2% [w/v] dextrose + 2% [w/v] agar). After inoculation on SNA and CMD media for 72 and 168 h, characteristics of the conidia, conidiophores, chlamydospores, and sori were examined and photographed under optical microscope (Olympus, BX53F) and stereoscopic microscopes (Olympus, SZX16), then compared with a published description [29].

For molecular identification, the genomic DNAs of *Trichoderma* isolate were extracted using the Cetyl Trimethyl Ammonium Bromide (CTAB) method as described by Stewart and Via [30] and the partial sequence of translation elongation factor 1-alpha gene (*tef1*) was amplified using the EF1–728 F/TEF1LLErev primer pair [31]. The PCR reaction mix were as follows: 1× Green TAP Mix, 0.2 μM primer EF1-728F, 0.2 μM primer TEF1LLErev, 100 ng gDNA. The *tef1* PCR was performed in a 96-well PCR instrument (Hangzhou, AGS, Medtech Co., LTD). The PCR products were purified using a ZTOPO-TA Fast Cloning Kit (Zomanbio, Kit No. ZC206–1), and sequenced by Guangzhou Tianyi Huiyuan Biotechnology Co., Ltd. (Guangzhou, China). The *tef1* sequences were deposited in GenBank (NCBI) to get accession number. The phylogenetic tree based on *tef1* sequences were construct in MEGA-X using the Neighbour-Joining method.

### In vitro evaluation of antagonistic activity of salt-tolerant Trichoderma isolate against F. oxysporum under salt stress

#### Dual culture and trypan blue staining

The antagonistic activity of the salt-tolerant *Trichoderma* isolate against the *F. oxysporum* isolate HGL.1 was evaluated by the dual culture method as described by Morton and Stroube [28] under salt stress of 100 mM NaCl or without. When cultured, the cover glasses were inserted into the dual culture plates to make the hyphae of *Trichoderma* and *F. oxysporum* grow onto them, and the plates were incubated at 28 °C. Seven days later, the radial growth radii of HGL.1 were recorded, and the interaction of hyphae on the cover glasses was observed under an optical microscope. Then mycelia in the dual culture plates were dyed using trypan blue staining method to determine whether the pathogen was killed by *Trichoderma* isolate [32], and the colour changing of the pathogen hypha was observed using optical microscope and photographed.

#### Effect of volatile and non-volatile inhibitors

The inhibitory effects of volatile and non-volatile metabolites produced by the salt-tolerant *Trichoderma* isolate against the *F. oxysporum* isolate HGL.1 were evaluated by the method described by Dennis and Webster [33, 34] in the presence of 100 mM NaCl or absent. To test the effect of volatile metabolites, a cellophane sheet was sandwiched between two petri dishes on which mycelium plugs of *Trichoderma* and *F. oxysporum* were inoculated, respectively. For non-volatile metabolites, *Trichoderma* was cultured on cellophane covering on the medium for 2 days and then *F. oxysporum* was inoculated on the medium after the cellophane was uncovered. The cultures were incubated at 28 °C in darkness for 7 days, and the colony radius of the pathogen was measured to calculate the inhibition ratio of mycelial growth.

### Effect of Trichoderma on alleviating salt stress of cucumber

Cucumber seeds (*Cucumis sativus* L.) of the cultivar Chuanlv 21 were surface-sterilized with 8% NaClO solution for 1 min, washed three times with distilled water, placed into distilled water for germinating, and then sown in plastic pots (10 cm calibre) with 0.5 kg composite soil (soil: sand = 7:3, one plant per pot). After 10 days, seedlings of uniform size were selected and the following treatments were compared: (1) 100 mL water + 100 mL spore suspension of *Trichoderma* (1 × 10^7^ cfu/mL) (marked as “T”); (2) 100 mL water + 100 mL water (marked as “control”); (3) 100 mL 100 mM NaCl solution+ 100 mL spore suspension of *Trichoderma* (1 × 10^7^ cfu/mL) (marked as “100S + T”); (4) 100 mL 100 mM NaCl solution+ 100 mL water (marked as “100S”); (5) 100 mL 200 mM NaCl solution+ 100 mL spore suspension of *Trichoderma* (1 × 10^7^ cfu/mL) (marked as “200S + T”); (6) 100 mL 200 mM NaCl solution+ 100 mL water (marked as “200S”). All treatments were arranged in completely randomized design (CRD), and at least 30 cucumber seedlings were used for each treatment. After 14 days, all the plants were harvested to measure their growth parameters and physiological-biochemical indexes.

#### Measurement of plant growth parameters

Twenty-one cucumber seedlings from each treatment were harvested randomly, rinsed for free of soil, and divided into roots and shoots. The length and fresh weight of roots and shoots were measured, and dry weights were recorded when all plant parts were dried to constant mass (115 °C for 30 min and 80 °C for 8 h).

#### Measurement of physiological-biochemical indexes

Nine cucumber seedlings from each treatment were harvested randomly, and the fresh leaves were collected for measuring the content of chlorophyll (Chl), soluble protein and malondialdehyde (MDA), and the activity of catalase (CAT) and peroxidase (POD).

The quantitative analysis of chlorophyll was done according to the method of Lichtenthaler and Wellburn [35]. The chlorophyll was extracted by acetone from 200 mg fresh leaves and the absorbance was read at 663- and 646- nm wavelength. The content of chlorophyll was calculated by the formula as follows:

$$\mathrm{Chl}\ \mathrm{a}\ \left(\mathrm{mg}/\mathrm{L}\right)=12.21\times {\mathrm{OD}}_{663}-2.81\times {\mathrm{OD}}_{646}$$

$$\mathrm{Chl}\ \mathrm{b}\ \left(\mathrm{mg}/\mathrm{L}\right)=20.13\times {\mathrm{OD}}_{646}-5.03\times {\mathrm{OD}}_{663}$$

$$\mathrm{Chl}\ \mathrm{total}\ \left(\mathrm{mg}/\mathrm{L}\right)=\mathrm{Chl}\ \mathrm{a}+\mathrm{Chl}\ \mathrm{b}$$

Chl content (mg/g) = (Chl. concentration × Extraction volume × dilution factor)/fresh weightLipid peroxidation was recorded in terms of concentration of malondialdehyde (MDA) by the method of Heath and Packer [36]. Absorbance at 450, 532 and 600 nm were used for the calculation of MDA equivalent. Blank sample was used as reference. MDA equivalent was calculated by the following equation:

$$\mathrm{MDA}\ \mathrm{equivalents}\ \left(\mathrm{nmol}/\mathrm{mL}\right)=6.45\times \left({\mathrm{OD}}_{532}-{\mathrm{OD}}_{600}\right)-0.56\times {\mathrm{OD}}_{450.}$$

The content of soluble proteins was measured according to the method described by Bradford [37]. Two hundred milligram fresh leaves were ground in sodium phosphate buffer (50 mM pH 7.0), and centrifuged at 4200 rpm (4 °C) for 10 mins. After centrifuging, the supernatant was collected in a 10 mL test tube, and 2 mL sodium phosphate buffer was added in the precipitate then centrifuged for the second time. Then mixed the supernatants and volumed to 10 mL. The protein extract is blue when reacted with Coomassie Brilliant Blue G-250, and the absorbance of the reaction solution at the wavelength of 595 nm was read. Standard curve of bovine serum albumin (BSA) was used as reference.

To analyse the activities of POD and CAT, 1.0 g fresh leaves were ground in 25 mL sodium phosphate buffer (50 mM pH 7.8) and centrifuged at 12000 rpm (4 °C) for 20 mins. The supernatant was collected to measure the activities of POD and CAT referred to the methods described by Samantary [38] and Kar and Mishra [39].

### Effect of Trichoderma on diminishing root rot of cucumber

Cucumber seeds (Chuanlv 21) were sown separately in plastic pots (17 cm in calibre, one seed per pot) containing 2 kg composite soil (soil: sand = 7:3, 4 plants per pot). When the third leaves emerged, one half of the seedlings were irrigated with 100 mL of 100 mM NaCl solution, and the other half were treated with water. The following treatments for the seedlings irrigated with salt solution or without were compared: (1) 100 mL spore suspension of *Trichoderma* (1 × 10^7^ cfu/mL) + 30 mL spore suspension of *F. oxysporum* (1 × 10^7^ cfu/mL) (marked as “T-Fo”); (2) 100 mL water + 30 mL spore suspension of *F. oxysporum* (1 × 10^7^ cfu/mL) (marked as “Fo”); (3) 100 mL water + 30 mL water (marked as “Control”). Before inoculated by pathogen, the cucumber roots were injured by inserting transplanting shovel into soil near the plant to damage the roots. All treatments were arranged in completely randomized design, and there were at least 40 cucumber seedlings for each treatment. The disease index and control effect of all 240 cucumber seedlings were assessed on 10 days post inoculation (dpi) using the grading standard of Li et al. [40]:

$$\mathrm{Disease}\ \mathrm{index}=\left[\Sigma\ \left(\mathrm{number}\ \mathrm{of}\ \mathrm{diseased}\ \mathrm{plants}\times \mathrm{rating}\ \mathrm{score}\right)/\left(\mathrm{total}\ \mathrm{number}\ \mathrm{of}\ \mathrm{plants}\times \mathrm{highest}\ \mathrm{rating}\ \mathrm{score}\right)\right]\times 100.$$

$$\mathrm{Control}\ \mathrm{effect}\ \left(\%\right)=\left[\left(\mathrm{infected}\ \mathrm{control}\ \mathrm{disease}\ \mathrm{index}-\mathrm{infected}\ \mathrm{treated}\ \mathrm{disease}\ \mathrm{index}\right)/\mathrm{infected}\ \mathrm{control}\ \mathrm{disease}\ \mathrm{index}\right]\times 100\%.$$

### Statistical analyses

Statistical analyses of quantitative data were performed using IBM SPSS Statistics 21 software (IBM Corp, Armonk, NY, USA) and included one-way or two-way analysis of variance (ANOVA) followed by Tukey’s test and Dunnett’s t-test at *P* < 0.05. Different superscript letters within the same column indicate statistically significant differences. All experiments were repeated at least three times.

## Results

### Isolation and identification of the pathogen causing root rot in cucumber

**Fig. 1:**
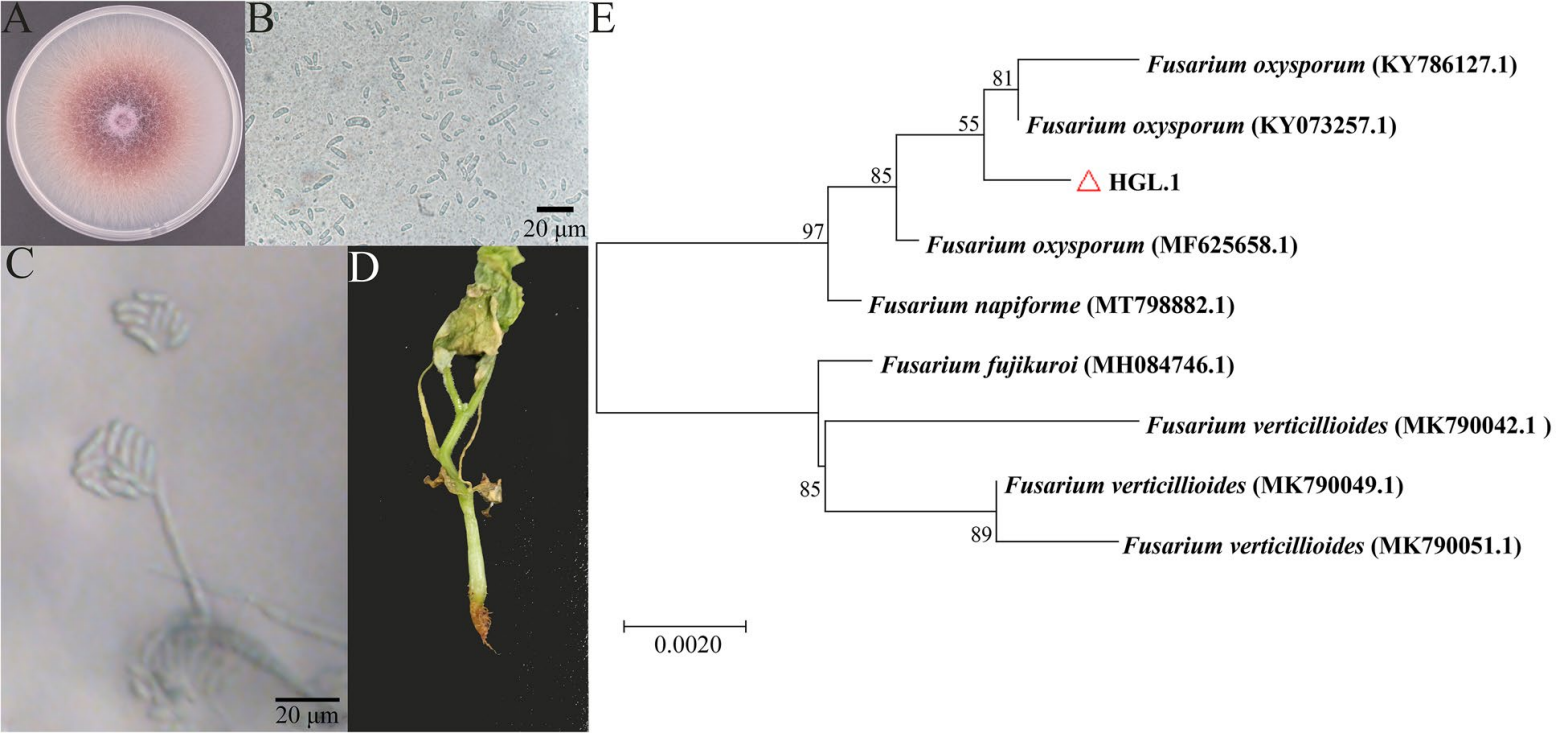
Identification of causal agent HGL.1 of cucumber root rot. **A** Morphology of colony inoculated on PDA for 7 days. **B, C** Characteristics of conidia and conidiophore. **D** Cucumber seedling infected by isolate. **E** Phylogenetic tree constructed in MEGA-X using the neighbor-joining method based on ITS

Ten isolates showing *Fusarium*-like morphological characteristics were obtained from diseased cucumber plants exhibited typical symptoms of root rot. Three isolates (HGL.1, HGL.2, and HGL.3) were randomly selected and cultured on PDA medium. The colonies of the three isolates initially showed white cotton flocculent on PDA medium and later became light red (Fig. 1A). The conidia were septate, sickle- or crescent-shaped, slightly curved or curved, and the two ends became tapered (Fig. 1B). The spores were attached to a long, tube-shaped conidiophore (Fig. 1C). These morphological features were consistent with previous descriptions of *F. oxysporum*. Pathogenicity test revealed that the plants inoculated showed rotted roots, wilted leaves and chlorosis at the leaf tips (Fig. 1D). The fungus can be re-isolated from the decaying tissue which was in accord with Koch’s postulates. An rDNA-ITS fragment of 513 bp was amplified and sequenced. The *ITS* sequence was deposited in GenBank and the accession number was MW091417. Phylogenetic analysis indicated that the isolate HGL.1 was closely related to *F. oxysporum* (GenBank Accession Nos. KY786127 and KY073257) (Fig. 1E). Therefore, the pathogen was identified as *F. oxysporum*.

### Trichoderma HN082102.1 showing salt tolerance and antagonistic activity against F. oxysporum

**Fig. 2:**
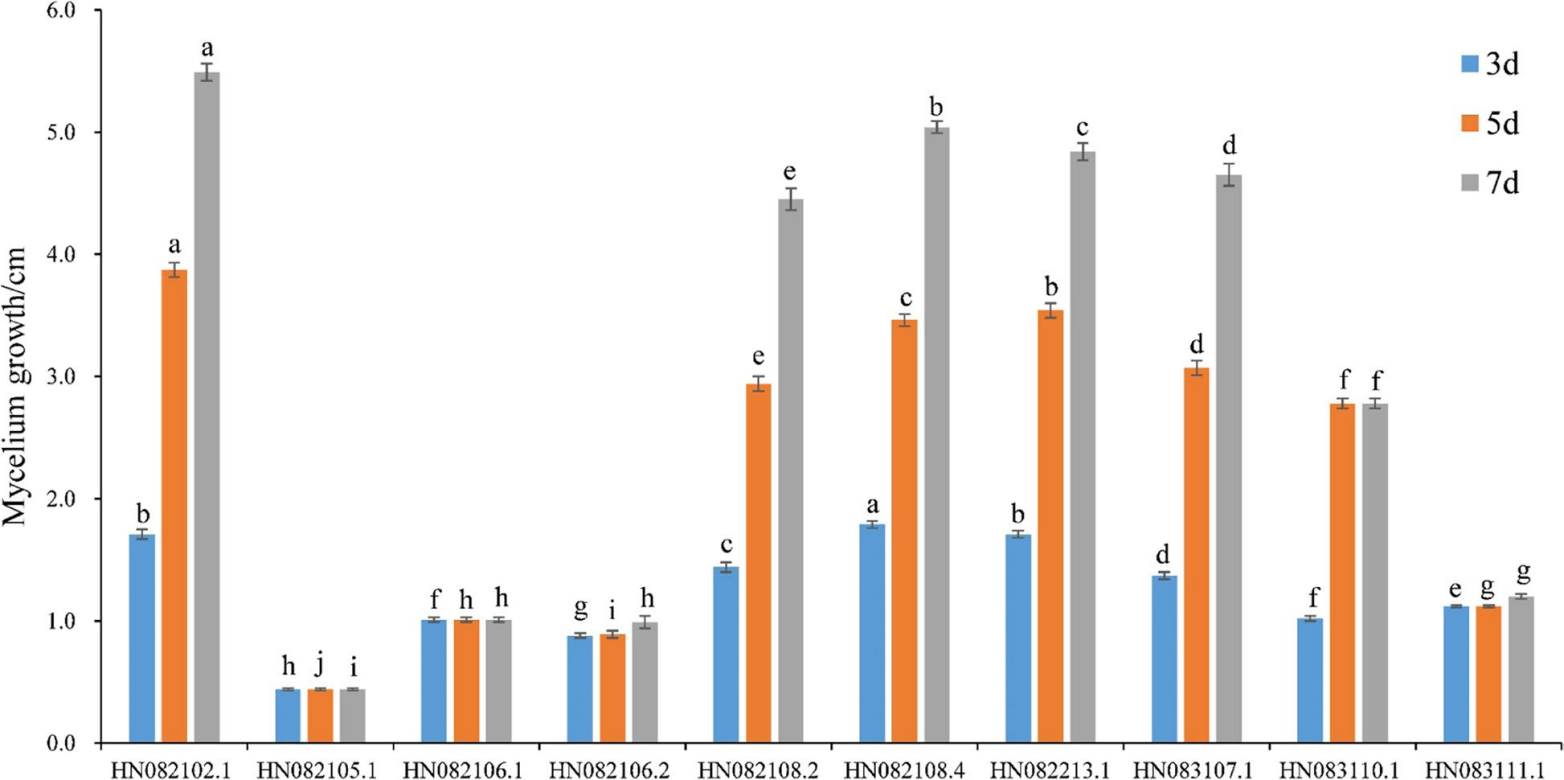
Colony diameter of 10 *Trichoderma* isolates inoculating on PDA contained 9% NaCl for 3, 5, 7 days. The lowercase letters indicate significant difference (*P* < 0.05) compared with the diameters measured on the same day

**Fig. 3:**
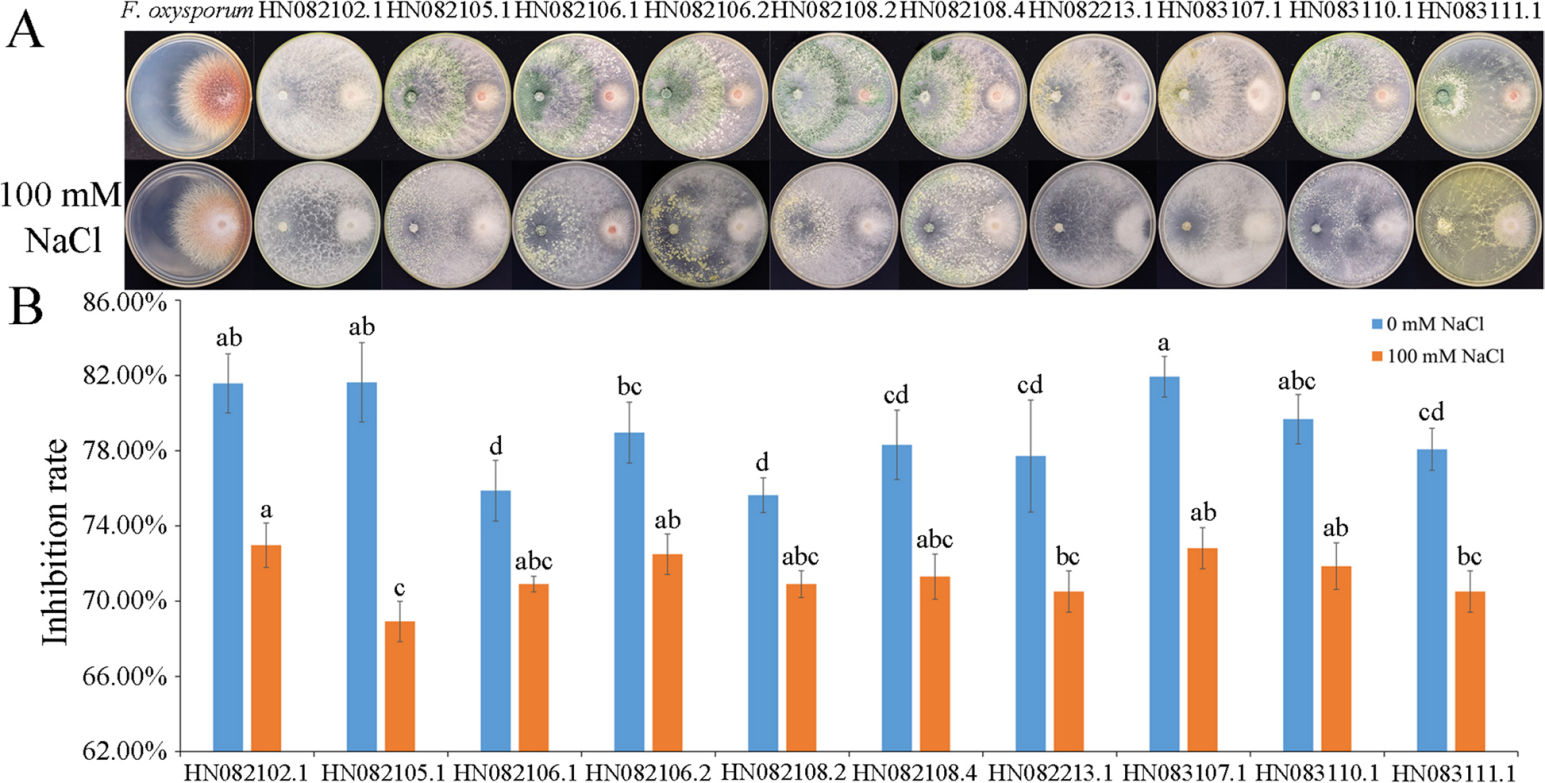
The antagonistic activity of the *Trichoderma* isolates that could grow on PDA medium contained 9% NaCl. **A** The dual culture of *Trichoderma* isolates and *F. oxysporum* HGL.1. **B** Inhibition rate of *Trichoderma* isolates to *F. oxysporum* HGL.1 with (100 mM NaCl) or without salt stress (0 mM NaCl). The lowercase letters indicate significant difference (*P* < 0.05) between the same treatment

Twenty-seven *Trichoderma* isolates were obtained from samples of 46 sea muds and 3 algae using dilution plate methods (Table 1). Among them, 27, 27, 22, and 10 isolates could grow on PDA amended with 3, 5, 7, and 9% (w/v) NaCl, respectively. Among the 10 isolates that could grow on the medium amended with 9% NaCl, HN082102.1 showed a particularly high level of salt tolerance, and its colony diameter was significantly bigger than others on the fifth and seventh day (Fig. 2). In addition, the isolate HN082102.1 also showed high antagonistic activity against *F. oxysporum* HGL.1 (Fig. 3). Therefore, HN082102.1 was chosen for further analysis.

**Table 1:** Source of *Trichoderma* isolates

Sample	Location	Strain code	***tef***1^a^
algae	Dongfang City, Hainan Province	HN082102.1, HN082102.2	MW133238, MW133239
sea muds	Dongfang City, Hainan Province	HN082104.1, HN082104.2, HN082105.1, HN082105.2, HN082106.1, HN082106.2	MW133240, MW133241, MW133242, MW133243, MW133244, MW133245
sea muds	Sanya City, Hainan Province	HN082108.2, HN082108.3, HN082108.4, HN082212.1, HN082213.1, HN082213.2, HN082213.3, HN082213.4, HN082213.5, HN082216.1	MW133246, MW133247 MW133248, MW133249, MW133250, MW133251, MW133252, MW133253, MW133254, MW133255
sea muds	Lingao City, Hainan Province	HN082323.1, HN082325.1, HN082328, HN083003.1, HN083107.1, HN083110.1, HN083111.1, HN090114.2, HN090118.1	MW133256, MW133257, MW133258, MW133259, MW133260, MW133261, MW133262, MW133263, MW133264

^a^GenBank accession numbers for translation elongation factor 1-alpha gene (*tef*1) partial sequence

### Taxonomic classification of Trichoderma strain HN082102.1

**Fig. 4:**
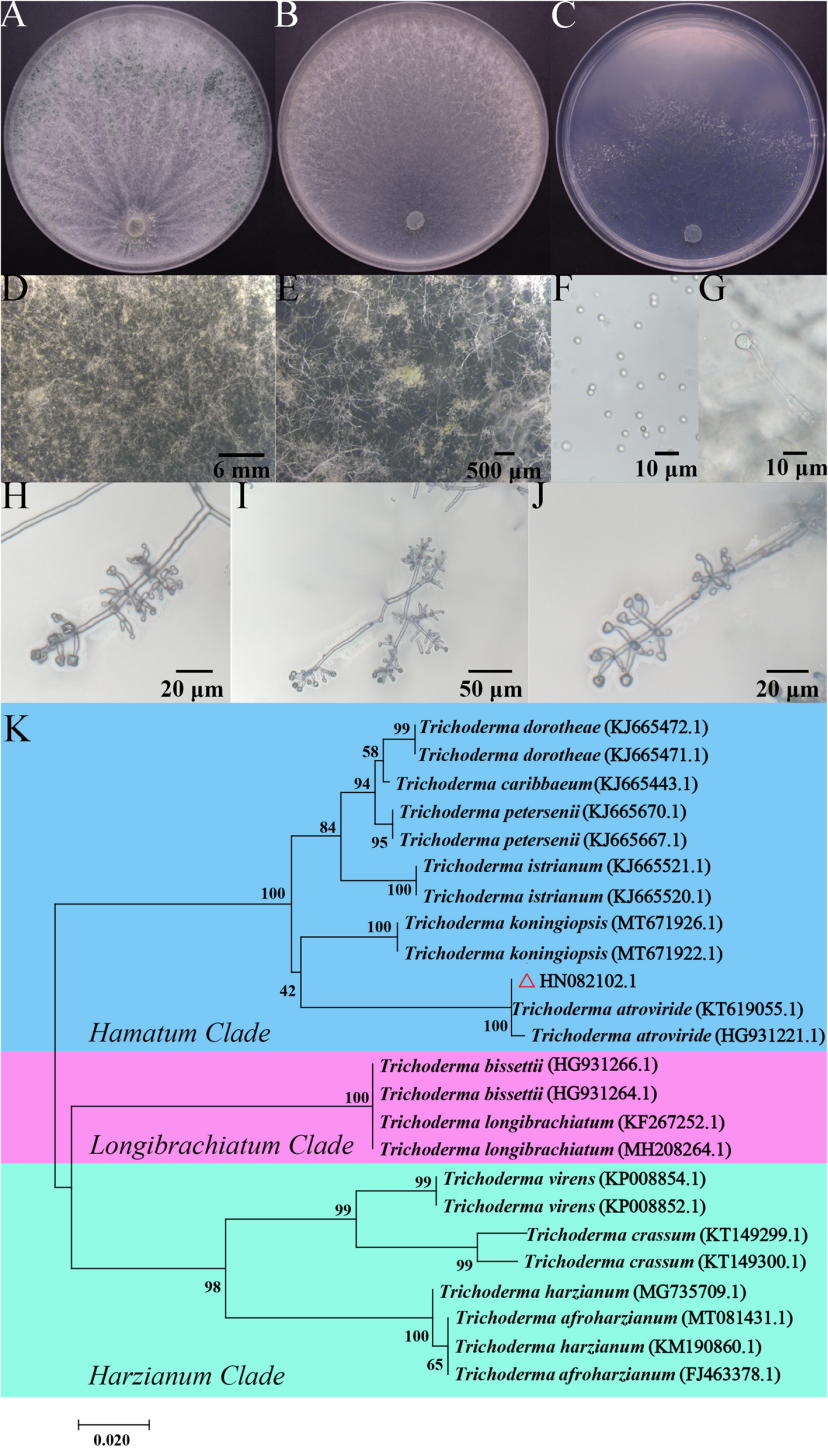
Identification of the isolate HN082102.1. **A-C** The colony morphology of HN082102.1 grown on PDA, CMD and SNA medium, respectively. **D, E** The sorus morphology of HN082102.1 grown on CMD medium. **F, G** Morphology of the conidia and chlamydospore produced by isolate HN082102.1. **H-J** The conidiophores of *Trichoderma* isolate HN082102.1. **K** Neighbour-joining tree showing the evolutionary relationships of different *Trichoderma* species based on translation elongation factor 1 (*tef1*) gene sequences

The isolate HN082102.1 produced green conidia on PDA medium and smelled of a distinctive coconut odour (Fig. 4A). Yellow green sori with cotton floc were observed on CMD medium (Fig. 4B, D, E), and cyan spores were distributed in a ring on the edge of SNA medium (Fig. 4C). The conidia were subspherical to ovoid with dimensions of 2.5–3.7 μm × 2.9–4.6 μm (Fig. 4F). The chlamydospores were abundant, globose to subglobose, and at the end or intercalary of hypha (Fig. 4G). The branches were of a pyramidal type with phialides holding in divergent whorls of three or four or arising singly near the tip of the main axis, but unilateral branches were common. Phialides were ampulliform when closely spaced, lageniform when more distantly spaced, straight or sinuous, sharply constricted at the neck when ampulliform, and 4.9–11.1 μm long and 2.1–8.5 μm wide at the widest point (Fig. 4H, I, J). These morphological characteristics were consistent with *T. atroviride* described by Bissett’s research. Phylogenetic analysis based on *tef1* sequence (accession number: MW133238) showed that isolate HN082102.1 was clustered with *T. atroviride* (HG931223.1, KT619055.1, and HG931221.1) (Fig. 4K). Therefore, isolate HN082102.1 was identified as *T. atroviride.*

### T. Atroviride HN082102.1 against F. oxysporum under salt stress

**Fig. 5:**
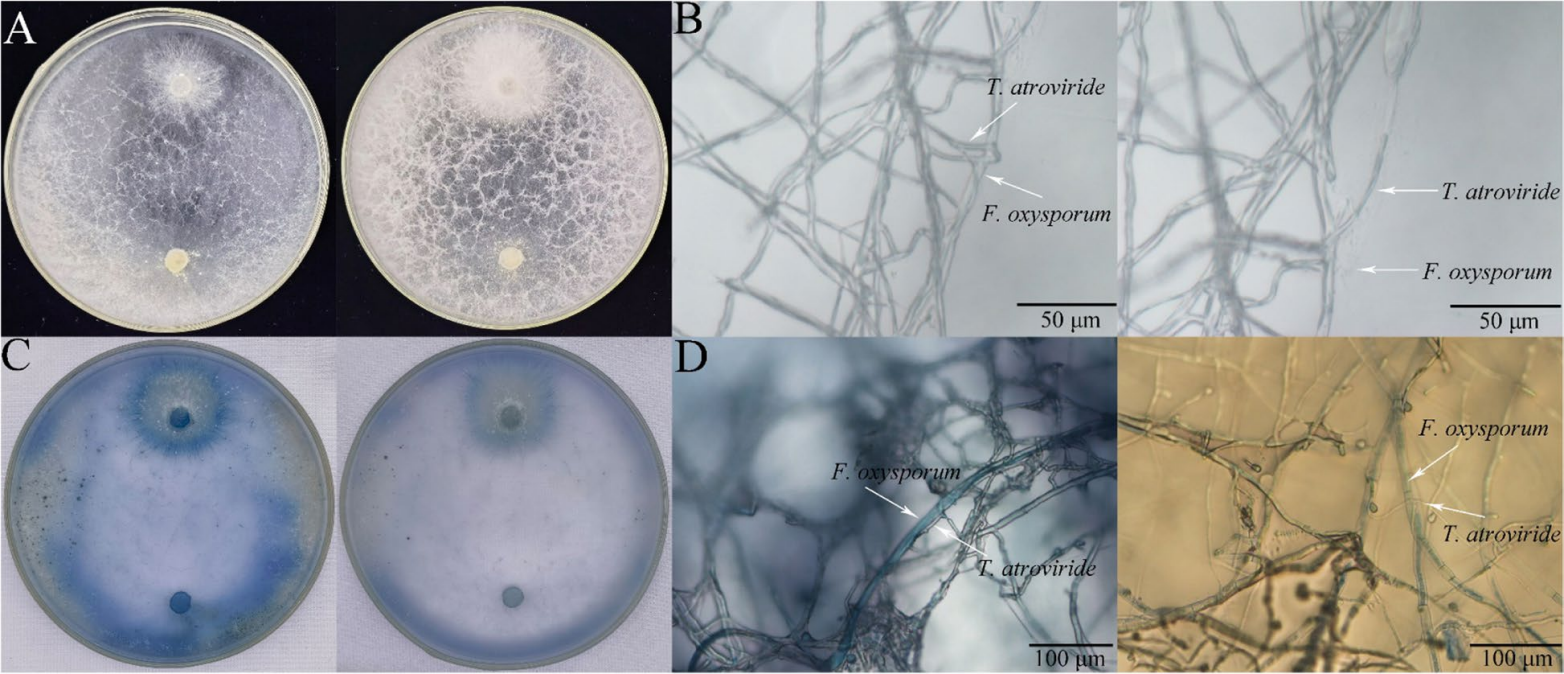
The interaction zone between *T. atroviride* HN082102.1 and *F. oxysporum* HGL.1. **A** Dual culture of *T. atroviride* and *F. oxysporum* in the presence (right) or absence (left) of 100 mM NaCl stress. **B** *T. atroviride* hyphae encircled *F. oxysporum* hyphae (left) and disintegrated hypha of *F. oxysporum* (right). **C** Trypan blue staining of *T. atroviride* and *F. oxysporum* in the presence (right) or absence (left) of 100 mM NaCl stress. **D** Stained *F. oxysporum* hyphae

*T. atroviride* HN082102.1 exhibited mycoparasitism to *F. oxysporum* HGL.1 causing root rot in cucumber in the presence or absence of 100 mM NaCl stress (Fig. 5A, B). The hyphae of *F. oxysporum* HGL.1 were encircled and disintegrated by *T. atroviride* HN082102.1 (Fig. 5B). Also, the hyphae of *F. oxysporum* at the interaction zone with *T. atroviride* were stained blue by trypan blue (Fig. 5C), indicating that these hyphae were killed. At the stained areas, the unstained *T. atroviride* hyphae adhered or entangled to the stained *F. oxysporum* hyphae (Fig. 5D).

**Fig. 6:**
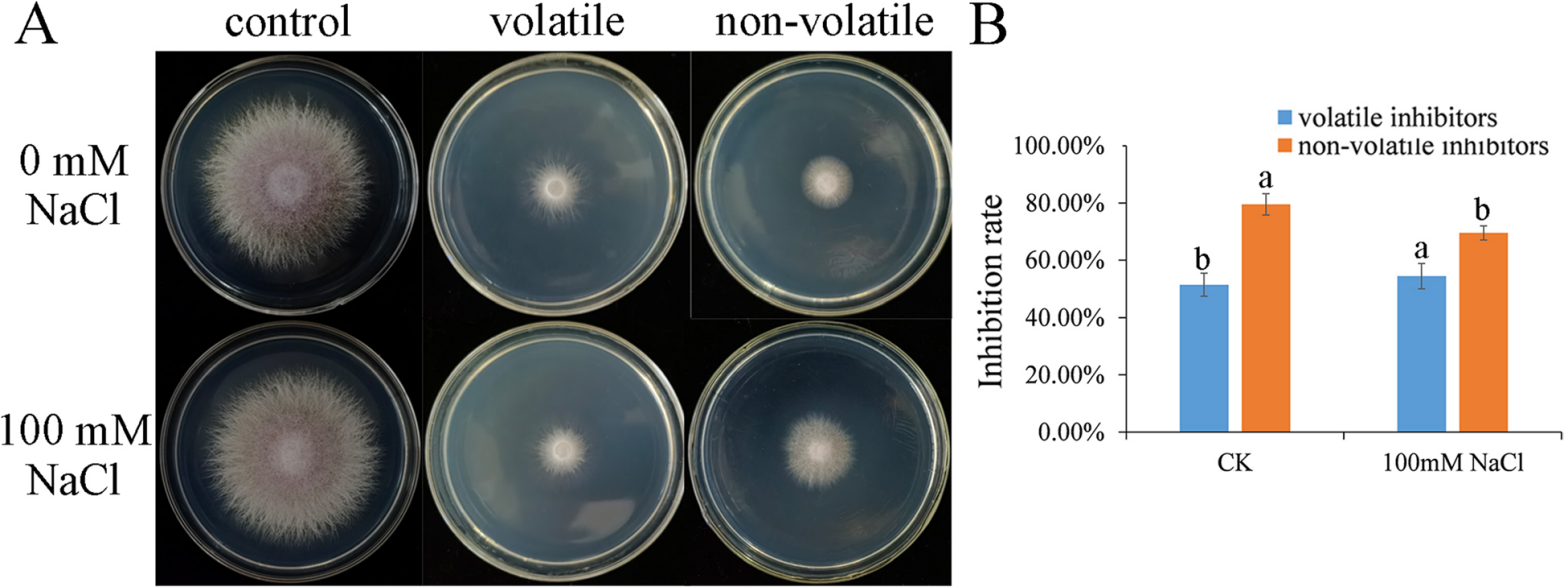
Effect of volatile and non-volatile inhibitors produced by *T. atroviride* HN082102.1 to *F. oxysporum* HGL.1 in the presence or absence of 100 mM NaCl stress. **A** *F. oxysporum* HGL.1 grown on PDA medium affected by volatile and non-volatile inhibitors. **B** The inhibition rate of volatile inhibitors and non-volatile inhibitors to the growth of *F. oxysporum* HGL.1. The lowercase letters indicate significant difference (*P* < 0.05) between the same treatment

The volatile and non-volatile metabolites produced by *T. atroviride* inhibited significantly the mycelium growth of *F. oxysporum*, and the inhibition rates were higher than 50%, even nearly 80% for non-volatile metabolites under no salt stress (Fig. 6). Interestingly, the inhibition rate of volatile metabolites to *F. oxysporum* increased by 3.00% under 100 mM salt stress compared with the no salt control, while for the non-volatile metabolites, it decreased by 9.96% (Fig. 6). It is suggested that salt stress may promote the inhibition effect of volatile substances to the pathogen, but show negative effect on the inhibition of non-volatile substances.

### T. Atroviride HN082102.1 alleviates the salt stress on cucumber growth

**Fig. 7:**
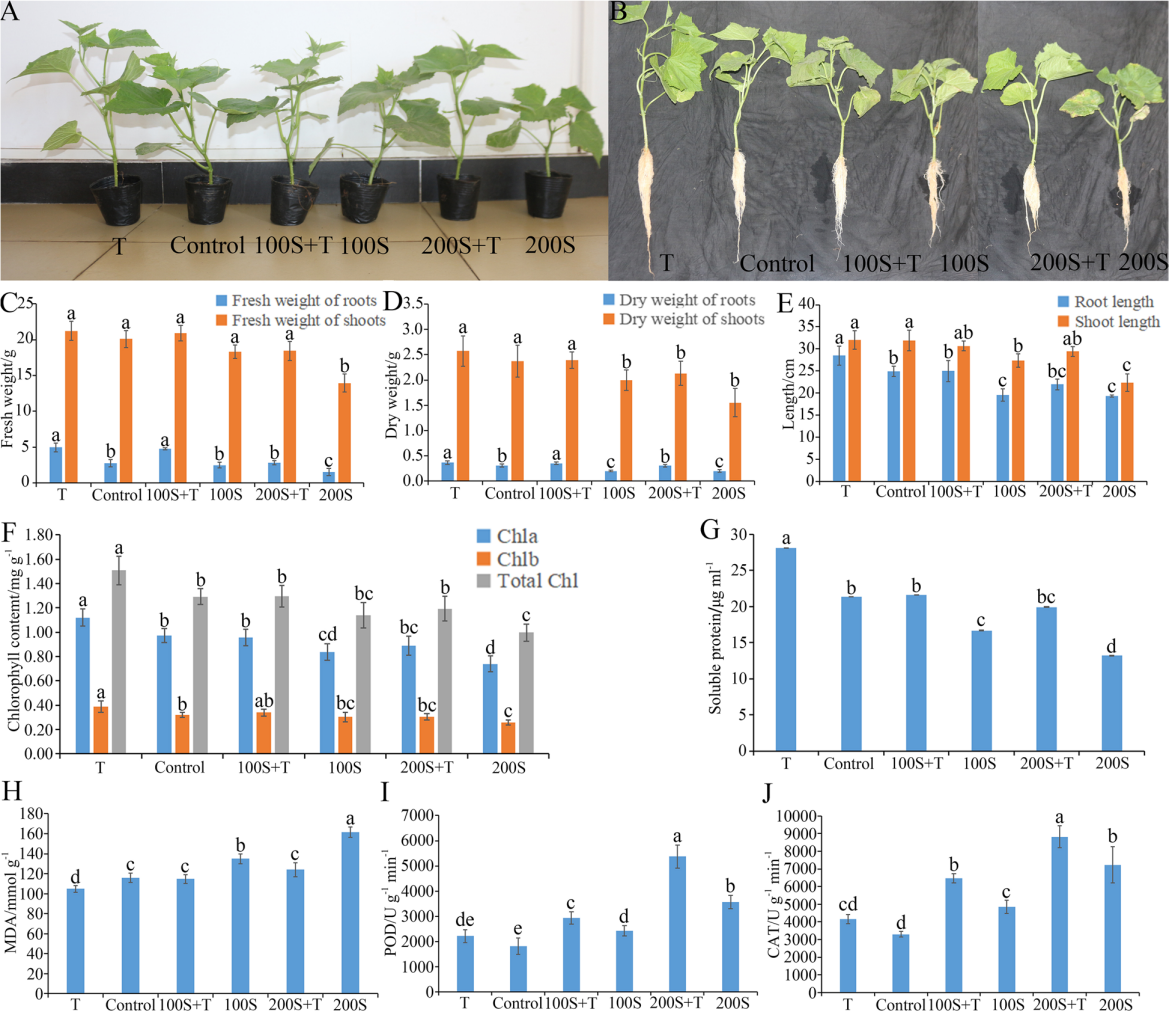
Effect of *T. atroviride* HN082102.1 on cucumber in the presence or absence of 100 mM NaCl stress. **A, B** The effect of *T. atroviride* HN082102.1 on growth of cucumber seedlings in the presence or absence of 100 mM NaCl stress. **C-E** The effect of *Trichoderma* on the fresh weight, dry weight, and length of roots and shoots of cucumber in the presence or absence of 100 mM NaCl stress. **F-J** The effect of *Trichoderma* on the content of chlorophyll, soluble protein and MDA, and the activity of POD and CAT in the presence or absence of 100 mM NaCl stress. “T” means “*Trichoderma* treated”; “Control” means “water control”; “100S” and “200S” mean “100 mM saline treatment” and “200mM saline treatment”, respectively; “100S + T” and “200S + T” mean “*Trichoderma* treated under 100 mM or 200 mM NaCl. The lowercase letters indicate significant difference (*P* < 0.05) between the same treatment

The application of *Trichoderma* mitigated the adverse effects of salt stress and promoted the growth of cucumber under 100 mM and 200 mM NaCl, especially for the root (Fig. 7A-E). The dry weight and length of roots reduced significantly when the cucumbers grew under 100 mM and 200 mM NaCl, also for the fresh weight of roots under 200 mM NaCl (Fig. 7C, D, E). When cucumbers were treated with *T. atroviride* HN082102.1 under 100 mM and 200 mM NaCl, root fresh weight increased by 92.55 and 84.86%, respectively, and root dry weight increased by 75.71 and 53.31%, respectively (Fig. 7E).

With NaCl increased (0, 100, and 200 mM), the total chlorophyll content of cucumber leaves decreased from 1.29 mg g^− 1^ to 1.00 mg g^− 1^. However, when *T. atroviride* HN082102.1 was applied, the content of total chlorophyll increased by 16.57, 13.93, and 19.68%, the content of chlorophyll a increased by 14.85, 14.66, and 20.38%, and the content of chlorophyll b increased by 21.80, 11.93, and 17.67% under 0 mM, 100 mM and 200 mM NaCl, respectively (Fig. 7F)*.*

The content of soluble protein of leaves decreased significantly by 21.92 and 38.26% under 100 mM and 200 mM NaCl compared with control, and increased by 29.48 and 51.23% when *Trichoderma* was used under 100 mM and 200 mM NaCl (Fig. 7G). The MDA content of leaves increased by 16.40 and 39.36% under 100 mM and 200 mM NaCl compared with control, and decreased by 14.94 and 23.14% when *Trichoderma* was used under 100 mM and 200 mM NaCl (Fig. 7H). The activities of antioxidant enzymes POD and CAT increased significantly under 100 mM and 200 mM NaCl compared with control, and increased more significantly when *Trichoderma* was applied, which were 20.94 and 33.23% under 100 mM NaCl and 50.78 and 21.83% under 200 mM NaCl (Fig. 7I, J). This suggests that POD and CAT may help cucumber to withstand NaCl stress.

What’s more, depending on the results above, the indexes above were not significantly different from or better than the control when *T. atroviride* was applied under 100 mM NaCl. So, 100 mM of NaCl was used in the following experiment.

### T. Atroviride HN082102.1 diminishes root rot of cucumber

**Fig. 8:**
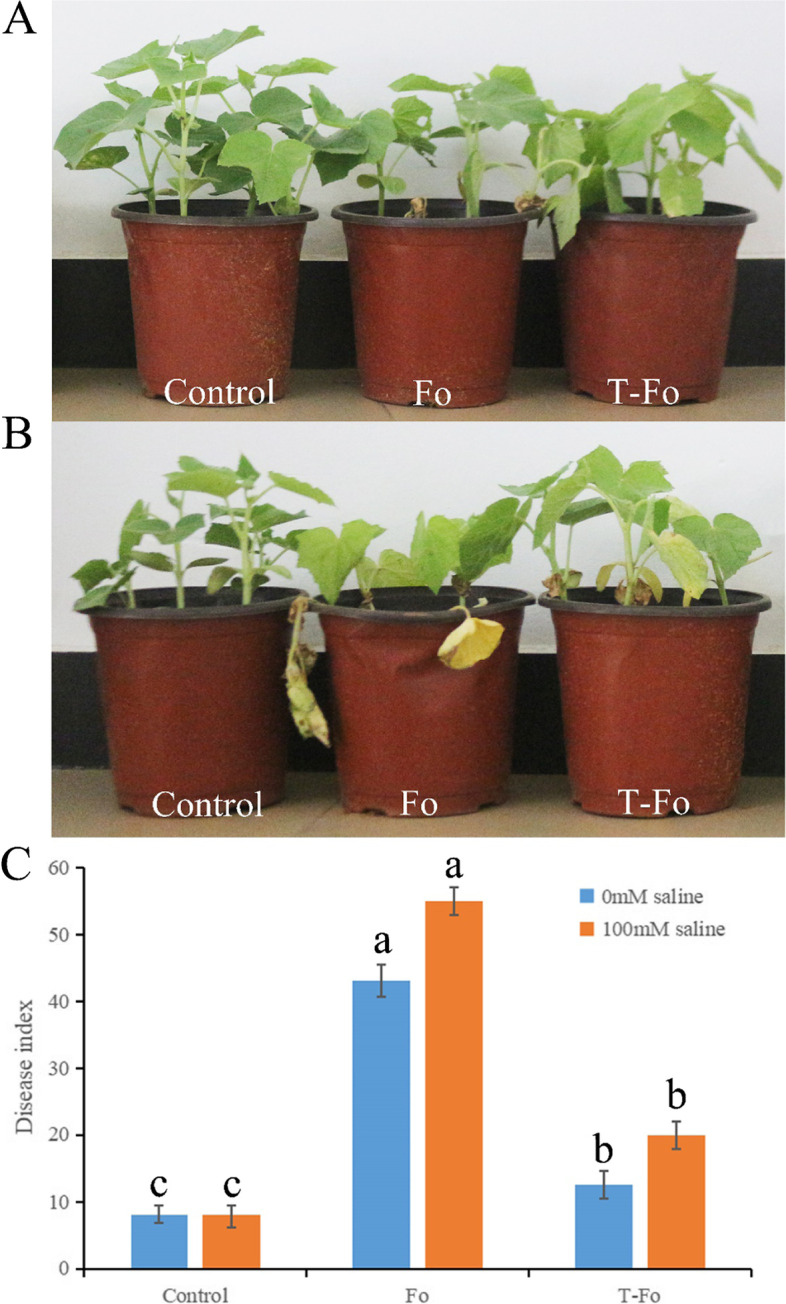
Control effect of *T. atroviride* HN082102.1 on cucumber root rot in greenhouse in presence and absence of 100 mM NaCl stress. **A** The symptoms of cucumber root rot with or without the application *Trichoderma* in the absence of NaCl. **B** The symptoms of cucumber root rot with or without the application *Trichoderma* in presence of 100 mM NaCl stress. **C** The effect of *T. atroviride* HN082102.1 to disease index of cucumber root rot in presence and absence of 100 mM NaCl stress. “Control” indicated with water; “Fo” indicated infected by *F. oxysporum* HGL.1; “T-Fo” indicated that before infected by *F. oxysporum* HGL.1, treated with *T. atroviride* HN082102.1. Data presented are the means ± SE. The lowercase letters indicate significant difference (*P* < 0.05) between the same treatment with two-way analysis of variance (ANOVA)

The *T. atroviride* isolate HN082102.1 could control root rot of cucumber under salt stress. In Greenhouse, when seedlings were treated with *T. atroviride* HN082102.1, cucumber root rot caused by *F. oxysporum* was significantly inhibited (Fig. 8). The disease index of cucumber root rot decreased from 43.125 to 12.5% in non-saline soil, while decreased from 55 to 20% in 100 mM NaCl saline soil (Fig. 8C). The control effect of isolate HN082102.1 on cucumber root rot was 71.01% in non-saline soil and was 63.64% in 100 mM NaCl saline soil.

## Discussion

In this study, 27 *Trichoderma* spp. were isolated from samples of 46 sea muds and 3 algae collected from marine habitats, and the salinity resistance and antagonistic activity of these isolates were examined. HN082102.1 isolated from algae exhibited the strongest salt tolerance and better antagonistic activity and therefore was selected to be used in the further research.

The growth parameters of cucumber, that are the length, fresh weight, and dry weight of shoots and roots, decreased significantly under NaCl stress, which might be attributed to increased osmotic stress, nutrient deficiencies, and disturbance of various physiological and biochemical mechanisms [41–43]. When the cucumber was treated with *T. atroviride* HN082102.1 under NaCl stress, the growth parameters increased, suggesting that *T. atroviride* may alleviate the adverse effect of salt stress to the cucumber growth. The findings were consistent with the results of Rawat et al. [44], which reported that *Trichoderma* isolates alleviated the negative impacts of salt stress in rice. The application of *Trichoderma* increased root length of plants, thereby promoting the plant to absorb nutrients and water from the soil and enhancing its ability to counteract salt stress [45, 46].

The application of *T. atroviride* HN082102.1 significantly increased the content of chlorophyll and soluble protein and reduced the MDA content in cucumber under salt stress. Previous studies also reported the similar results [16, 47]. Zörb et al. [48] concluded that the decreased content of chlorophyll under salt stress was related to the negative effects of salt stress on chloroplasts. Furthermore, Sultana et al. [49] demonstrated that this phenomenon was due to the increased activity of chlorophyll-degrading enzymes, thus reduced net chlorophyll synthesis. Zhao and Zhang [50] reported that the application of *Trichoderma* to plants under salt stress increased IAA and GA levels and decreased ABA levels. Martínez-Medina et al. [51] and Resende et al. [52] also found that plant hormones like GA, ABA, and CTK play an important role in enhancing chlorophyll content. Therefore, the increased content of chlorophyll associated with *Trichoderma* application under salt stress may be related to its effect on plant hormones. The increased soluble protein content associated with *Trichoderma* application was probably beneficial in reducing the osmotic potential of plant cells [53], thereby enabling the plant to absorb water from the external saline solution under salt stress. The reduction of MDA content is an important stress tolerance index, as reported in mulberry [54] and chickpea [55]. An increased MDA content reflected membrane damage caused by lipid peroxidation in the presence of ROS [56]. Exogenous application of *Trichoderma* reduced salt stress in cucumber, which may be attributed to reduce ROS production in treated plants.

The activity of the antioxidant enzymes POD and CAT increased under salt stress, and after the application of *Trichoderma* their activity increased more. To prevent the membrane damage caused by O^2−^ and H_2_O_2_, POD reduces H_2_O_2_ to water [57], and CAT transforms H_2_O_2_ to water and molecular oxygen [58]. Increased activity of antioxidant enzyme means that more ROS can be removed, thereby reducing ROS damage to plants. The application of *Trichoderma* increased the activity of antioxidant enzymes and was therefore conducive to scavenging more ROS produced under salt stress, thus reducing stress-related damage.

With or without salt stress, the application of *T. atroviride* had a significant inhibition effect on cucumber root rot, which was consistent to the reports described by Rawat et al. [59] and Kashyap et al. [13]. Singh et al. [60] demonstrated that tomato plants treated with *Trichoderma* obtained tolerance toward root rot by upregulating the activities of POD, polyphenol oxidase, phenylalanine ammonia lyase, and by increasing the content of total phenols. Likewise, Bae’s [59] study demonstrated that phenolic glucoside levels increased significantly after the application of *Trichoderma* in cucumber. These results suggest that metabolites released by *Trichoderma* isolates not only act directly on the pathogen but also trigger the plant to release defense-related compounds. Here, it was found that the effect of isolate HN082102.1 on cucumber root rot was somewhat decreased in saline soil, which may be caused by a decline in *Trichoderma* metabolites. Anyway, this is the first report of salt-tolerant *T. atroviride* isolated from marine habitat can alleviate salt stress and diminish cucumber root rot caused by *F. oxysporum*. Taken together, *T. atroviride* HN082102.1 can be used as biological control agent in saline alkali land.

## Conclusion

This is the first report of salt-tolerant *T. atroviride* isolated from marine habitat showing antagonistic activity to *F. oxysporum*. The application of *T. atroviride* HN082102.1 markedly increased tolerance to salt stress and reduced the severity of root rot caused by *F. oxysporum* under saline conditions in cucumber. The increasing of ROS scavenging and maintaining the osmotic balance are key observations of the ability to tolerate salt. Therefore, *T. atroviride* strain HN082102.1 has the potential to improve plant growth and disease resistance serving as biological control agent in saline alkali land.

## Data Availability

Not applicable.

## Abbreviations

ROS: Reactive Oxygen Species
PODs: Peroxidases
ITS: Internal Transcribed Spacer
PDA: Potato Dextrose Agar
mPDA: Modified Potato Dextrose Agar
SNA: Synthetic Low Nutrient Agar
CMD: Corn Meal Dextrose
CTAB: Cetyl Trimethyl Ammonium Bromide
tef1: Translation Elongation Factor 1-Alpha gene
Chl: Chlorophyll
MDA: Malondialdehyde
CAT: Catalase
BSA: Bovine Serum Albumin
